# Which is the Optimal Frozen Elephant Trunk? A Systematic Review and Meta-Analysis of Outcomes in 2161 Patients Undergoing Thoracic Aortic Aneurysm Surgery Using E-vita OPEN PLUS Hybrid Stent Graft *versus* Thoraflex™ Hybrid Prosthesis

**DOI:** 10.21470/1678-9741-2019-0220

**Published:** 2020

**Authors:** Amer Harky, Matthew Fok, Mohamad Bashir

**Affiliations:** 1 Department of Cardiothoracic Surgery, Liverpool Heart and Chest Hospital, Liverpool, United Kingdom.; 2 Department of Vascular Surgery, Royal Liverpool Hospital, Liverpool, United Kingdom.; 3 Vascular Surgery Department, Royal Blackburn Teaching Hospital, Haslingden Rd, Blackburn, United Kingdom.

**Keywords:** Aortic Aneurysm, Thoracic, Tocopherols, Cardiopulmonary Bypass, Stents, Morbidity

## Abstract

**Objective:**

To systematically review the rate of morbidity and mortality associated with the use of E-vita hybrid stent graft and ThoraflexTM in patients undergoing complex aortic surgery.

**Methods:**

A comprehensive search was undertaken among the four major databases to identify published data about E-vita or Thoraflex™ in patients undergoing repair of thoracic aortic aneurysms.

**Results:**

In total, 28 papers were included in the study, encompassing a total of 2,161 patients (1,919 E-vita and 242 Thoraflex™). Patients undergoing surgery with E-vita or Thoraflex™ were of similar age and sex. The number of patients undergoing non-elective repair with Thoraflex™ was higher than with E-vita (35.2% *vs*. 28.7%, respectively). Cardiopulmonary bypass time was associated with increasing mortality in E-vita patients, however a meta-analysis of proportions showed higher 30-day mortality, permanent neurological deficit, and one-year mortality for Thoraflex™ patients. Direct statistical comparisons between E-vita and Thoraflex™ was not possible due to heterogeneity of studies.

**Conclusion:**

Although there are limited studies available, the available data suggests that mortality and morbidity are lower for the E-vita device in thoracic aortic aneurysm surgery than for Thoraflex™. Long-term data of comparative studies do not yet exist to assess viability of these procedures.

**Table:** 

Abbreviations, acronyms & symbols	
CI	= Confidence interval
COPD	= Chronic obstructive pulmonary disease
CPB	= Cardiopulmonary bypass
EVAR	= Endovascular aneurysm repair
HTN	= Hypertension
IHD	= Ischemic heart disease
MeSH	= Medical Subject Headings
PRISMA	= Preferred Reporting Items for Systematic Reviews and Meta-Analyses
TEVAR	= Thoracic endovascular aortic repair

## INTRODUCTION

The introduction of the elephant trunk technique by Borst et al. in 1983 facilitated the arch and distal aortic aneurysm repair in two stages^[1]^. The first stage was entailed ascending and aortic arch replacement through median sternotomy, while at the second stage, a free-floating conventional elephant trunk resultant of an extension of the arch prosthesis was left behind in the proximal descending aorta^[2,3]^.

In 1996, the development of a new elephant trunk prosthesis facilitated the treatment of aortic arch and proximal descending pathologies in a single operation. This was aimed at minimizing complications associated with the two-staged conventional elephant trunk. This new elephant trunk was known as “the frozen elephant technique”. The new device promoted the hybrid approach in which the endovascular component can be performed either simultaneously or in stages. The “hybrid” vascular graft made up of a conventional tube graft with an endovascular stented graft at the distal end was used to achieve a blood-tight seal in the descending aorta^[4-6]^. This permitted dual interventions, the aortic arch and the proximal descending pathologies, in a single operation. The latter was aimed at minimizing complications and reducing the high mortality associated with the classical elephant technique^[7,8]^.

The advent of this new device technology in prosthesis design from both commercially available devices, the JOTEC- E-vita (JOTEC GmbH, Hechingen, Germany) and the Thoraflex™ (VASCUTEK, Terumo, Inchinnan, Scotland, United Kingdom) prostheses, necessitated an appraisal of published outcomes. This systematic review and meta-analysis aims to evaluate the latest evidence regarding outcomes from the aforementioned devices.

## METHODS

### Literature Search Strategy

Electronic database searches were performed with MEDLINE, Google Scholar, Ovid, and Scopus from inception to December 2017. Limits were placed on manuscripts written in the English language only. Search terms used included Thoraflex, Evita, frozen elephant trunk, FET, aortic hybrid procedures, and thoracic hybrid procedures.

To achieve maximum sensitivity, all search terms were combined with Boolean operators and searched as both keywords and Medical Subject Headings (MeSH) terms. Following exclusion of articles based on title or abstract, full text articles selected had reference lists searched for any potential further articles to be included in this review.

### Selection Criteria

Studies in which patient cohorts underwent thoracic aortic surgery with frozen elephant trunk, with either E-vita device or Thoraflex™, in any type of pathology, including type A chronic dissection with residual disease, chronic type B dissection, and aneurysmal disease, were included. Studies were excluded if they included a paediatric population, case reports, or small case series, reviews, or editorials. When institutions published duplicate studies with accumulating numbers of patients or increased lengths of follow-up, only the most complete reports were included for quantitative assessment at each time interval.

### Data Extraction and Critical Appraisal

Data was extracted by two independent reviewers, and if necessary, a third was consulted to resolve disagreements. The information was extracted from studies that had met the inclusion criteria: baseline demographics and preoperative characteristics, operative urgency, extent of disease, cardiopulmonary bypass (CPB) time, cross-clamp time, type of brain protection strategy used, length of stay, postoperative stroke/paraplegia, and in-hospital mortality and mortality at one year, five years, and 10 years. The quality of evidence from each study was assessed using the Moose system.

### Statistical Analysis

Standard descriptive statistics (reported as means with 95% confidence intervals [CI]) were used to summarize demographic and baseline data of the recruited patients from all eligible studies. Meta-analyses of outcomes when reported were performed on the reported incidence of 30-day mortality, in-hospital stroke, and one-year mortality. Relative risk was used as summary. Heterogeneity among studies was estimated with χ^2^ tests, which was reported as the I2 statistic to estimate the percentage of total variation across studies, due to heterogeneity rather than chance.

Dependant on the heterogeneity determined, a fixed or a random effect model was used. All statistical analyses were conducted with the Review Manager software (Cochrane Collaboration, Software Update, Oxford, United Kingdom), version 5.1.2.

## RESULTS

### Baseline Demographics

**Fig. 1:**
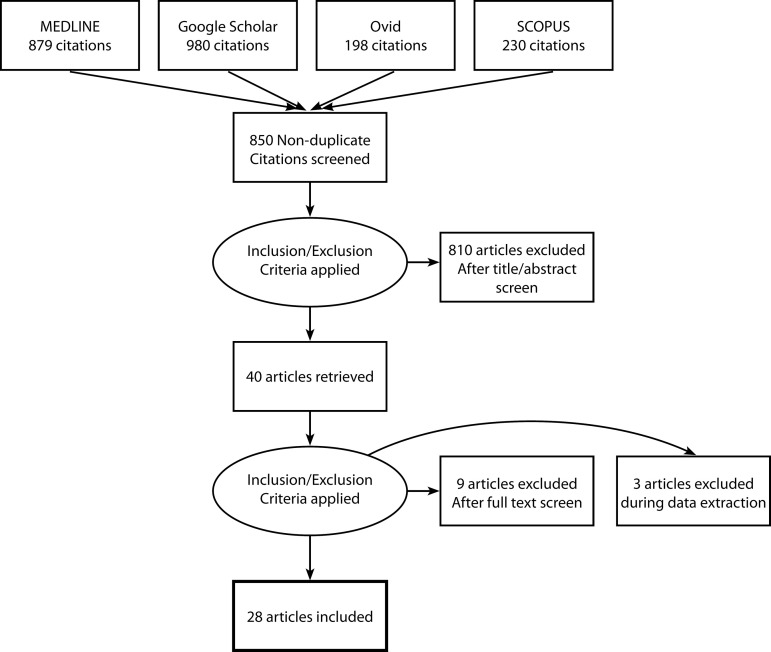
Preferred Reporting Items for Systematic Reviews and Meta-Analyses (PRISMA) chart.

In total, 28 papers were included in the study^[9-37]^, all were published after 2008, and they comprised a total of 2,161 patients (1,919 E-vita and 242 Thoraflex™) (Figure 1). Patients undergoing surgery with E-vita or Thoraflex™ were of similar age and sex (mean age 61.0 *vs*. 61.3, respectively; male percentage 70%) and the number of patients undergoing emergency repair with Thoraflex™ was higher than with E-vita (35.2% *vs*. 28.7%, respectively). Other baseline characteristics including diabetes, hypertension, ischaemic heart disease, previous stroke, previous cardiac surgery, renal disease, or Marfan syndrome were recorded poorly and are available in Table 1. Operative urgency (elective, emergency, mixed), aneurysm pathology (aneurysm, dissection, mixed), and extent of the disease pathology (ascending arch, descending arch, and descending) are available in Table 2.

**Table 1:** Baseline demographics.

**E-vita**				**Demographics**			**Preoperative characteristics**								
**No**	**Year**	**Author**	**N**	**Mean age**	**Male** **(n)**	**Female (n)**	**Diabetes** **(n)**	**HTN** **(n)**	**COPD** **(n)**	**IHD** **(n)**	**Smoking (n)**	**Previous stroke/neurological deficit (n)**	**Previous cardiac surgery (n)**	**Previous TEVAR/EVAR (n)**	**Renal disease (n)**
1	2013	Ius et al.	30	61.0	24	6	n/a	22	3	2	5	5	6	n/a	3
2	2014	Verhoye et al.	16	59.3	13	3	n/a	n/a	2	n/a	n/a	3	5	0	1
3	2013	Mestres et al.	113	67.0	73	40	16	n/a	20	32	n/a	9	23	10	10
4	2013	Tsagakis et al.	132	59.0	95	37	n/a	n/a	n/a	n/a	n/a	n/a	16	3	n/a
5	2012	Gorlitzer et al.	3	58.0	1	2	n/a	n/a	n/a	n/a	n/a	n/a	n/a	3	n/a
6	2011	Di Eusanio et al.	49	59.6	43	6	1	46	2	4	n/a	n/a	39	1	4
7	2011	Pacini et al.	90	57.0	72	18	n/a	n/a	10	8	n/a	3	62	5	8
8	2015	Weiss et al.	57	58.0	42	15	5	n/a	10	6	n/a	3	21	10	5
9	2013	Hoffman et al.	32	58.0	26	6	n/a	n/a	n/a	3	n/a	4	n/a	n/a	1
10	2009	Di Bartolomeo	34	61.7	29	5	n/a	28	n/a	n/a	16	n/a	19	n/a	4
11	2011	Tsagakis et al.	106	57.0	88	24	5	83	21	n/a	n/a	5	38	n/a	n/a
12	2009	Di Bartolomeo et al.	24	62.4	21	3	n/a	17	n/a	n/a	9	n/a	9	n/a	4
13	2010	Di Bartolomeo et al.	67	61.1	55	12	9	58	4	n/a	n/a	2	36	n/a	6
14	2014	Di Eusanio et al.	21	65.6	18	3	3	13	5	n/a	6	n/a	6	n/a	0
15	2008	Zipfel et al.	126	64.0	89	37	n/a	n/a	n/a	n/a	n/a	n/a	n/a	n/a	n/a
16	2013	Leontyev et al.	51	64.0	27	24	9	27	6	n/a	n/a	n/a	9	n/a	n/a
17	2017	Verhoye et al.	94	64.0	62	32	21	79	20	n/a	n/a	3	47	4	19
18	2013	Leontyev et al.	46	69.0	23	23	7	36	3	n/a	n/a	n/a	8	0	0
19	2013	Di Eusanio et al.	122	61.0	106	16	3	106	19	n/a	n/a	n/a	69	n/a	3
20	2014	Di Eusanio et al.	19	58.0	18	1	n/a	n/a	n/a	n/a	n/a	n/a	4	n/a	n/a
21	2015	Leontyev et al.	509	64.1	357	152	45	420	95	n/a	n/a	29	144	32	n/a
22	2016	Jakob et al.	178	59.0	125	53	16	n/a	41	n/a	n/a	22	61	n/a	n/a
**Thoraflex^TM^**				**Demographics**			**Preoperative characteristics**								
**No**	**Year**	**Author**	**N**	**Mean age**	**Male (n)**	**Female (n)**	**Diabetes (n)**	**HTN (n)**	**COPD (n)**	**IHD (n)**	**Smoking (n)**	**Previous stroke/neurological deficit (n)**	**Previous cardiac surgery (n)**	**Previous TEVAR/EVAR (n)**	**Renal disease (n)**
1	2012	Shrestha et al	34	60.0	25	9	n/a	n/a	n/a	n/a	n/a	2	10	0	0
2	2013	Ius et al	35	61.0	24	11	n/a	22	0	9	8	2	9	n/a	5
3	2017	Di Marco et al	44	n/a	n/a	n/a	n/a	n/a	n/a	n/a	n/a	n/a	n/a	n/a	n/a
4	2017	Landau et al	15	n/a	n/a	n/a	n/a	n/a	n/a	n/a	n/a	n/a	n/a	n/a	n/a
5	2016	Wong et al	14	57.0	11	3	n/a	14	n/a	n/a	n/a	n/a	n/a	n/a	n/a
6	2016	Shrestha et al	100	59.0	65	35	n/a	n/a	n/a	n/a	n/a	n/a	28	n/a	17

COPD=chronic obstructive pulmonary disease; EVAR=endovascular aneurysm repair; HTN=hypertension; IHD=ischemic heart disease; TEVAR=thoracic endovascular aortic repair

**Table 2:** Disease characteristics.

		E-vita	Thoraflex^TM^
Operative urgency*	Elective	718 (71.2%)	92 (64.8%)
	Non-elective	290 (28.7%)	50 (35.2%)
Pathology*	Aneurysm	578	57
	Dissection	1239	112
	Mixed	92	0
Extent of aneurysm*	Ascending	427	42
	Arch	109	11
	Descending	57	0
	Arch and descending	916	0

* Data not available in all studies.

**Fig. 2:**
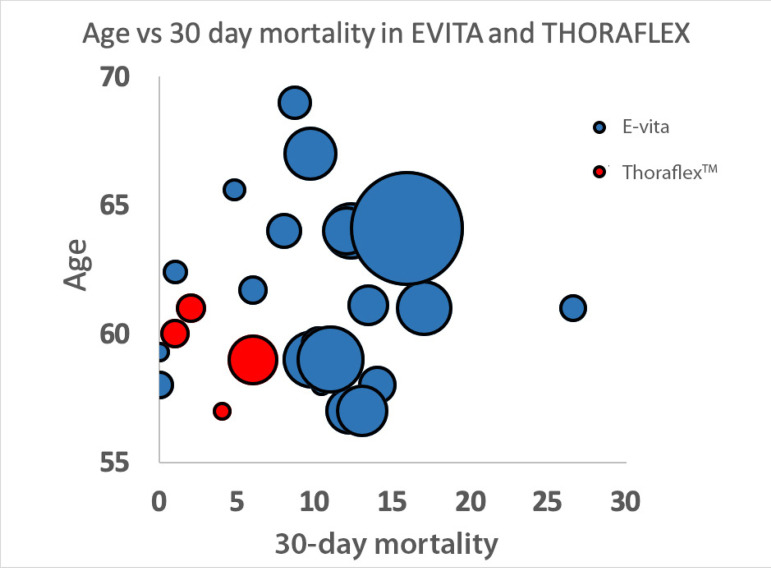
Bubble chart displaying the average age of patients in each study against 30-day mortality. E-vita and ThoraflexTM studies are represented by the colours blue and red, respectively (the bubble’s size is weighted with patient years of follow-up of each study, studies from the same centre that may represent similar or overlapping patients were removed to avoid duplication).

**Fig. 3:**
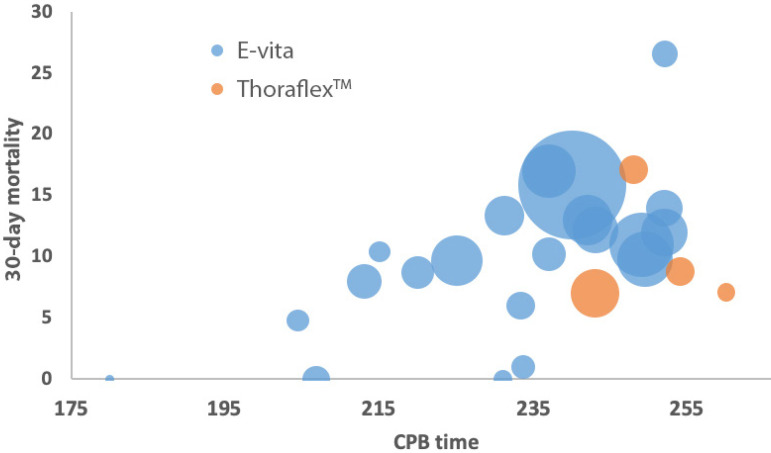
Bubble chart displaying the average cardiopulmonary bypass (CPB) time of patients in each study against 30-day mortality. E-vita and ThoraflexTM studies are represented by the colours blue and orange, respectively (the bubble’s size is weighted with patient years of follow-up of each study, studies from the same centre that may represent similar or overlapping patients were removed to avoid duplication).

Age is represented in Figure 2 by a bubble chart showing the relationship between 30-day mortality and average age in studies with E-vita and Thoraflex™. CPB is represented by a bubble chart that shows that increasing CPB is associated with an increase in 30-day mortality, particularly in E-vita studies (Figure 3).

### Thirty-day Mortality

**Fig. 4:**
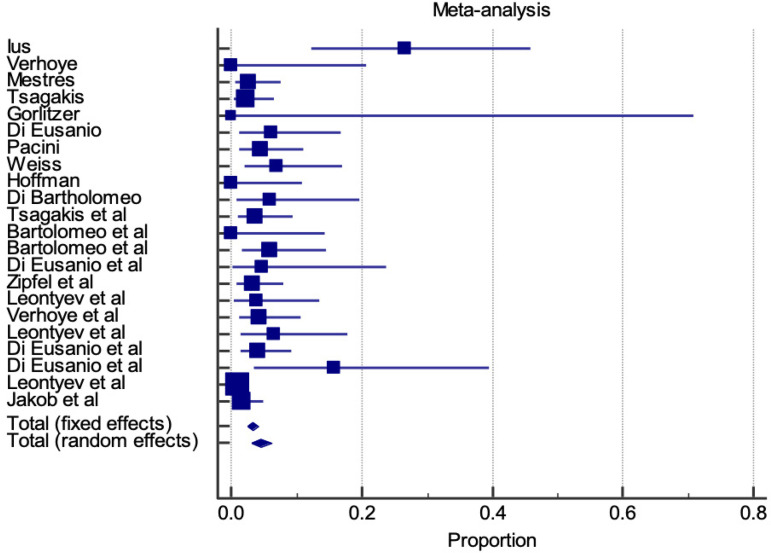
Forest plot displaying proportional meta-analysis plot for 30-day mortality with E-vita. Fixed effect (3.295 95% CI, 2.546 to 4.189), I2 56.97% (95% CI, 30.78 to 73.25). CI=confidence interval.

**Fig. 5:**
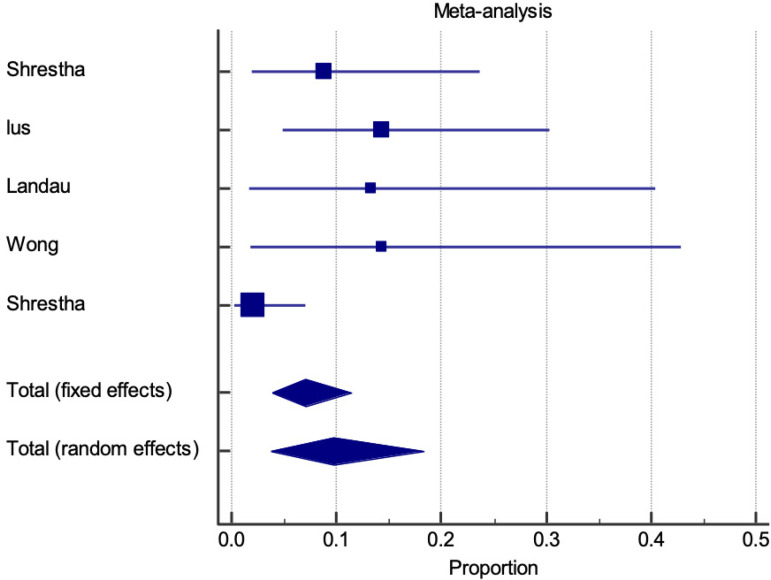
Forest plot displaying proportional meta-analysis plot for 30-day mortality with ThoraflexTM. Fixed effect (6.988 95% CI, 3.891 to 11.412), I2 61.78% (95% CI, 0.00 to 85.61). CI=confidence interval.

Thirty-day mortality is represented by a proportional meta-analysis plot for E-vita and Thoraflex™ studies (Figures 4 and 5, respectively). Data was available in 22 papers for E-vita and four papers for Thoraflex™.

**Fig. 6 A and B:**
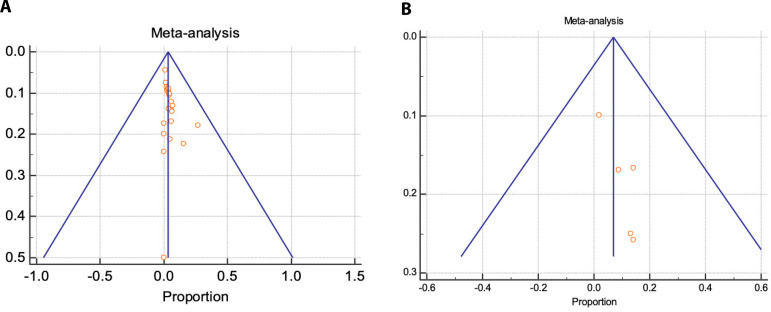
Publication bias assessment via funnel plots for 30-day mortality with E-vita and ThoraflexTM, respectively.

Proportional meta-analysis revealed a higher 30-day mortality with Thoraflex™ than with E-vita: pooled proportion, fixed effect (3.295 95% CI, 2.546 to 4.189), I2 56.97% (95% CI, 30.78 to 73.25) with E-vita *vs*. pooled proportion, fixed effect (6.988 95% CI, 3.891 to 11.412), I2 61.78% (95% CI, 0.00 to 85.61) with Thoraflex™. Funnel plots for both Thoraflex™ and E-vita proportional meta-analyses are shown in Figures 6A and 6B, respectively.

### In-hospital Stroke

**Fig. 7:**
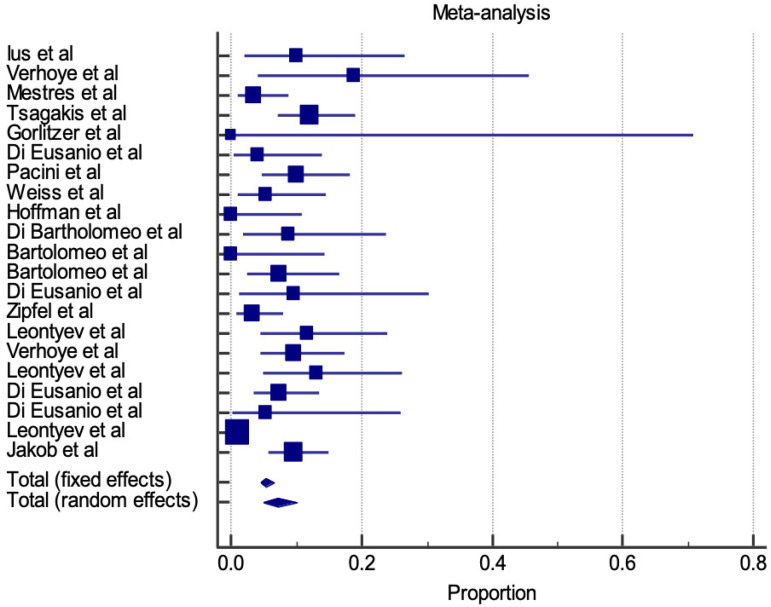
Forest plot displaying proportional meta-analysis plot for in-hospital stroke with E-vita. Fixed effect (5.417 95% CI, 4.426 to 6.554), I2 74.09% (95% CI, 60.24 to 83.12). CI=confidence interval.

**Fig. 8:**
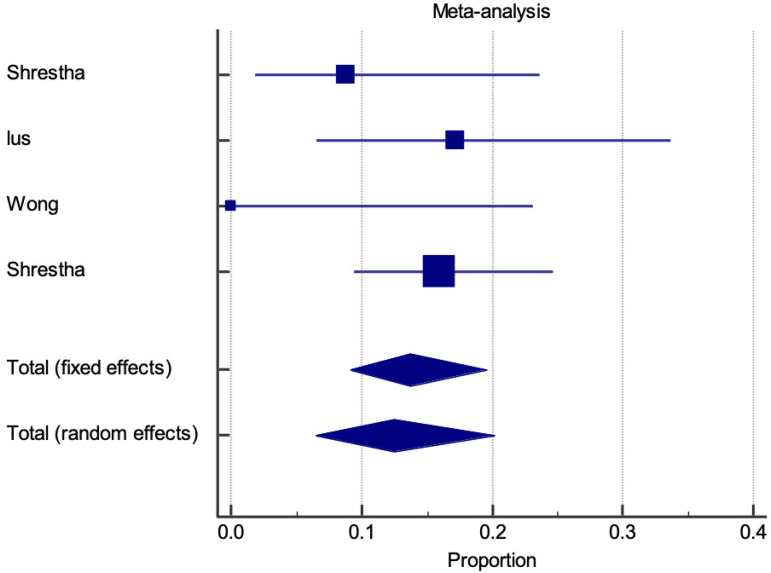
Forest plot displaying proportional meta-analysis plot for in-hospital stroke with ThoraflexTM. Fixed effect (13.737 95% CI, 9.149 to 19.517), I2 42.74% (95% CI, 0.00 to 80.78). CI=confidence interval.

**Fig. 9 A and B:**
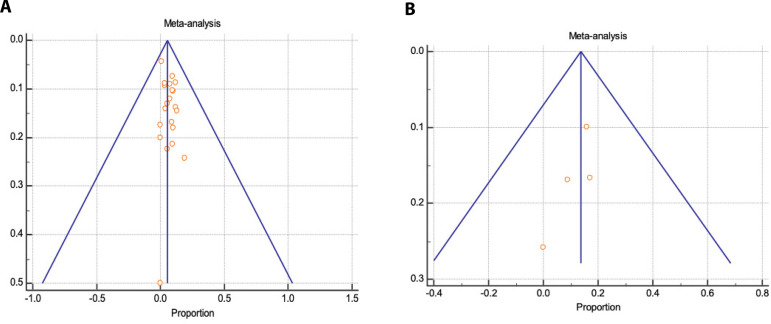
Publication bias assessment via funnel plots for in-hospital stroke with E-vita and ThoraflexTM, respectively.

In-hospital stroke is represented for both E-vita and Thoraflex™ by a forest plot proportional meta-analysis (Figures 7 and 8). Data was available in 22 papers for E-vita and four papers for Thoraflex™. Proportional meta-analysis revealed a higher in-hospital stroke rate with Thoraflex™ than with E-vita: pooled proportion, fixed effect (5.417 95% CI, 4.426 to 6.554), I2 74.09% (95% CI, 60.24 to 83.12) with E-vita *vs*. pooled proportion, fixed effect (13.737 95% CI, 9.149 to 19.517), I2 42.74% (95% CI, 0.00 to 80.78) with Thoraflex™. Funnel plots for both E-vita and Thoraflex™ proportional meta-analyses are shown in Figures 9A and 9B, respectively.

### One-year Mortality

**Fig. 10:**
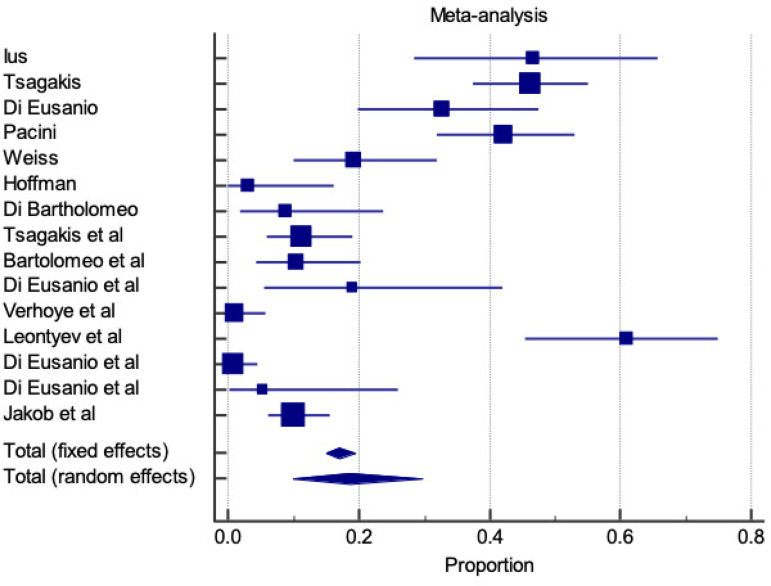
Forest plot displaying proportional meta-analysis plot for one-year mortality with E-vita. Fixed effect (17.041 95% CI, 14.856 to 19.405), I2 94.42% (95% CI, 92.24 to 95.98). CI=confidence interval.

**Fig. 11:**
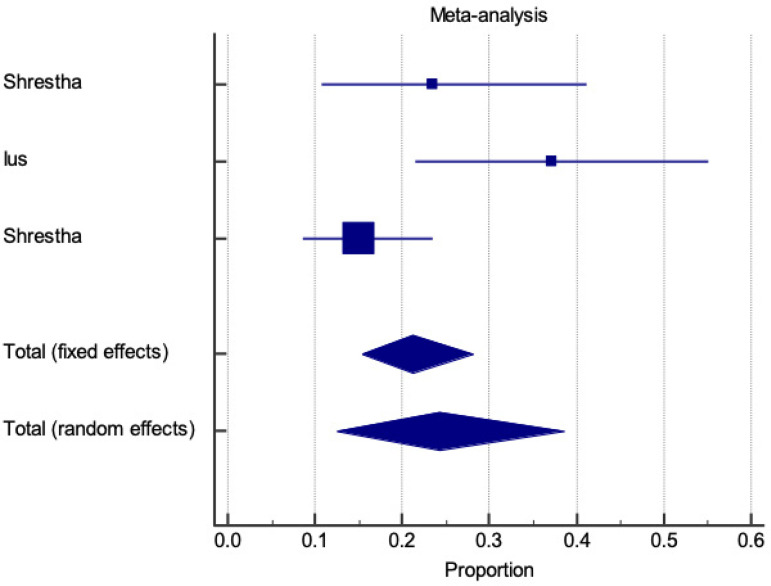
Forest plot displaying proportional meta-analysis plot for one-year mortality with ThoraflexTM. Fixed effect (21.25 95% CI, 15.392 to 28.127), I2 72.28% (95% CI, 6.39 to 91.79). CI=confidence interval

**Fig. 12 A and B:**
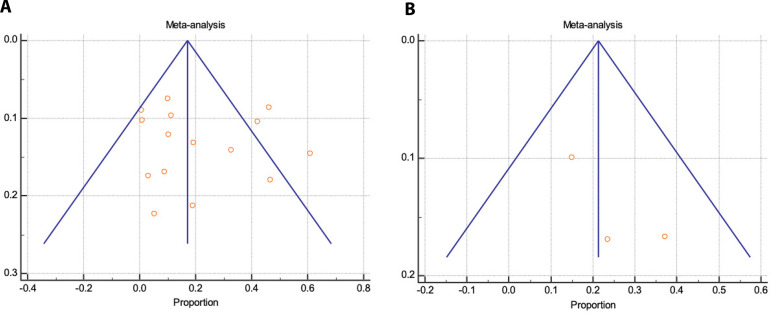
Publication bias assessment via funnel plots for one-year mortality with E-vita and ThoraflexTM, respectively.

One-year mortality is represented for both E-vita and Thoraflex™ by a forest plot proportional meta-analysis (Figures 10 and 11). Data was available in 17 papers for E-vita and three studies for Thoraflex™. One-year mortality was higher with Thoraflex™ than with E-vita: pooled proportion, fixed effect (17.041 95% CI, 14.856 to 19.405), I2 94.42% (95% CI, 92.24 to 95.98) with E-vita *vs*. pooled proportion, fixed effect (21.25 95% CI, 15.392 to 28.127), I2 72.28% (95% CI, 6.39 to 91.79) with Thoraflex™. Funnel plots for both E-vita and Thoraflex™ proportional meta-analyses are shown in Figures 12A and 12B, respectively.

## DISCUSSION

The conventional elephant trunk technique requires a two-stage operation. Each stage is associated with its own mortality and morbidity risks. The first-stage mortality rate ranges from 0% to 32.1%^[35,36]^, and second stage ranges from 0% to 33.3%^[35,36]^. However, the frozen elephant technique, which promoted a single stage aortic repair, represented a mortality ranging from 0% to 12.8%^[37]^. In two studies that compared conventional and frozen elephant trunk techniques, the rate of in-hospital deaths was similar among both techniques; the mean in-hospital mortality for elephant trunk technique was 21.6% and 13.9%^[22,26]^ and for frozen elephant trunk technique it was 8.7%^[25]^ and 4.8%^[26]^ (*P* 0.100)^[22,26]^. However, it becomes crucial to delineate the type of device used, the indications, and pathology. The advent of VASCUTEK Thoraflex™ Hybrid prosthesis meant that both home and commercially available devices had to be lined up for comparison based on outcomes and false lumen thrombosis. In this review, we looked at every study that utilized frozen elephant trunk and as intended we exposed the device used and compared the outcomes published.

Tian et al.^[37]^ in a previous meta-analysis focused on the safety and efficacy of the frozen elephant trunk technique. They deduced in their analysis, which included 17 observational studies, a pooled mortality of 8.3%, stroke rate of 4.9%, and spinal cord injuries rate of 5.1%. Amongst their studies, only five studies reported the five-year survival to be between 63% and 88%.

They also reported a strong linear correlation of length of time for CPB, myocardial ischemia, and circulatory arrest with perioperative mortality. When we looked into this microscopically, CPB time was associated with increasing mortality in the E-vita group, however meta-analysis of proportions showed a higher 30-day mortality and one-year mortality for Thoraflex™. This paradox surely could be explained by the fact that the number of patients undergoing emergency repair with Thoraflex™ was higher than those with E-vita. Alternatively, another thought for this paradox is based on a rather hard to quantify concept regarding the understanding and application of device technology used in multiple aortic extent operations. The fact that E-vita was launched and used previously to Thoraflex™ allowed us as surgeons to appreciate the concept of frozen elephant trunk device and ability to interpret and make decisions on procedural extent and surgical applications.

In a meta-analysis done by a group from Taiwan^[37]^ on the efficacy and safety of the frozen elephant technique in the setting of acute type A aortic dissection, they reported an overall in-hospital mortality rate of 8%. The authors indicated clearly in their analysis that based on their findings the frozen elephant technique does not bring unacceptable mortality or morbidity risks for treating acute type A aortic dissection aggressively with frozen elephant trunk. Hence, if we dissect this further, we note that Thoraflex™ Hybrid prosthesis was clearly utilized more frequently in emergency settings across the studies we pooled and that it clearly represents a focal point – that this device despite its wide use in emergency setting had rather less efficacious and safe proviso. Compounding this further, the permanent neurological deficit that we found was higher amongst the Thoraflex™ group. Surely and again, this is true because the device was being used rather more frequently in the emergency setting. This further raises another intriguing point regarding the aggressiveness and extent of the aortic segment replaced during a given emergency setting. One could argue this based on the fact that results could be potentially skewed owing to publication bias, however, as shown in Figure 6A, the sensitivity and publication bias analyses for the E-vita device were rather indicative that the findings were robust; none of the study overly influenced the findings and there was no publication bias. However, for the Thoraflex™ group, this was equivocal (Figure 6B). We think this was potentially due to the scarcity in outcome reporting on studies utilizing Thoraflex™ device. This was seen again in figures depicting in-hospital stroke between the two devices. Surely, we can deduct from this that the evidence of the robustness of Thoraflex™ use and the judgement regarding its supremacy over the former E-vita device are questionable and opened to many interpretations.

In their meta-analysis review on hybrid arch techniques to provide a safe alternative to open repair, a group from Athens^[38]^ reported that acceptable short- and mid-term results remain debatable, with immediate need for prospective trials to compare open conventional techniques with the hybrid methods using hybrid device prosthesis with or without endovascular methods.

## CONCLUSION

The implementation of new surgical techniques offers chances but carries risks. The frozen elephant trunk technique has increasingly been used to treat complex aortic pathologies of the aortic arch and the descending aorta, however, it’s prudent to elicit as this review demonstrated that there is still an ongoing discussion regarding the optimal frozen elephant trunk use and its indications. Although there are limited studies available, the available data suggests that mortality and morbidity were lower for the E-vita device on surgeries in thoracic aortic aneurysm surgery.

### Limitations

There are several limitations to our analysis that should be considered when interpreting the results. The number of comparative studies included in our analysis was too small to perform a comparison of the frozen elephant technique with other techniques to treat type A aortic arch dissection. Direct statistical comparisons between E-vita and Thoraflex™ was not possible due to heterogeneity of studies. Most of the included studies were retrospective in design and differences in surgical parameters and patient baseline characteristics may partially explain the heterogeneity seen across the studies and possibly affect our results. There was little information in the included studies regarding long-term survival, indicating the need for well-designed long-term studies to investigate this question.

**Table:** 

Author's roles & responsibilities	
AH	Substantial contributions to the conception or design of the work; or the acquisition, analysis, or interpretation of data for the work; drafting the work or revising it critically for important intellectual content; final approval of the version to be published
MF	Substantial contributions to the conception or design of the work; or the acquisition, analysis, or interpretation of data for the work; drafting the work or revising it critically for important intellectual content; final approval of the version to be published
MB	Substantial contributions to the conception or design of the work; or the acquisition, analysis, or interpretation of data for the work; drafting the work or revising it critically for important intellectual content; final approval of the version to be published
